# State-of-the-art management of locally advanced head and neck cancer

**DOI:** 10.1038/sj.bjc.6602510

**Published:** 2005-04-20

**Authors:** T Y Seiwert, E E W Cohen

**Affiliations:** 1 Department of Medicine, Section of Hematology/Oncology, Pritzker School of Medicine, The University of Chicago, Chicago, IL 60637-1470, USA; 2 Cancer Research Center, University of Chicago, Pritzker School of Medicine, The University of Chicago, Chicago, IL 60637-1470, USA

**Keywords:** head & neck cancer, chemoradiotheraphy, squamous cell carcinoma (HNSCC), epidermal growth factor receptor (EGFR)

## Abstract

During the past 20 years, treatments for head and neck squamous cell carcinoma (HNSCC) have changed dramatically owing largely to the advent of novel approaches such as combined modality therapy as well as improvements in surgical and radiotherapeutic techniques. Locally advanced disease in particular, which engendered very high recurrence and mortality rates, is now associated with long-term disease-free survival in the majority of cases. This article will focus on locally advanced HNSCC, which frequently remains a clinical challenge, review state-of-the-art therapy, and introduce promising novel therapies. The field continues to evolve rapidly with new evidence during the past year clearly establishing the benefit of adjuvant chemoradiotherapy (CRT), as well as early evidence showing improved survival with the use of an epidermal growth factor receptor inhibitor in combination with radiotherapy. There are varied regimens in use for patients with locally advanced disease, but at the same time the multitude of options can plague the clinician when trying to select the most appropriate one. This article will attempt to put the various approaches into perspective and propose an evidence-based treatment algorithm.

Head and neck cancer has an estimated annual global incidence of 533 100 cases (Parkin *et al*, 2001) and is the fifth most common cancer worldwide with the great majority of cases being squamous cell carcinomas (HNSCC). There is strong geographical variance in incidence likely related to associated risk factors, with the highest reported rates being observed in some areas of France (Bas-Rhin, male incidence 63.58 cases/100 000 people) and India/central Asia (Sankaranarayanan *et al*, 1998). The staging for HNSCC is shown in Table 1. Treatment for locally advanced disease (stages III, IVA, IVB), which makes up more than 50% of all cases, requires aggressive and concerted measures, and oftentimes remains a clinical challenge. Until recently, 5-year survival rates were reported to be below 30% for patients with stage IVA/B disease (Vokes *et al*, 1993) and 40% for all locally advanced tumours (Laramore *et al*, 1992) even with early multimodality approaches (30% recur locally, 25% distally).

## PERSPECTIVE

Current management of locally advanced HNSCC (Table 1) has evolved from poorly effective single modality therapy to an integrated, highly effective multidisciplinary approach. Unlike early stage HNSCC, all three modalities – surgery, radiotherapy, and chemotherapy – play vital and complementary roles. The various combination treatments have led to several competing approaches, each with distinct advantages and disadvantages, and initial treatment can vary significantly between institutions. Therefore, the following paragraphs will examine each major approach, highlight important differences and advantages/disadvantages, and attempt to recommend a management flowchart based on the evidence. Treatments used for locally advanced disease can be classified as follows:

- Surgery followed by adjuvant chemoradiotherapy (CRT) (or radiation)
- CRT upfront (with surgery as a salvage treatment)
- Induction chemotherapy followed by definitive local therapy:
  - CRT
  - Other primary treatment options: radiotherapy, surgery (±adjuvant therapy)
- epidermal growth factor receptor (EGFR) inhibition in combination with radiotherapy or CRT

### Postoperative therapy: surgery followed by adjuvant CRT (or radiation)

Predictors of recurrence after surgical resection include involved margins of resection, extranodal/extracapsular spread, perineural invasion, and the presence of two or more involved regional lymph nodes (Snow *et al*, 1982; Vikram *et al*, 1984; Kramer *et al*, 1987). Since locoregional failures remained the dominant problem, adjuvant locoregional therapies such as radiation and subsequently CRT were added and adjuvant therapy is now considered standard of care for stage III/IVA/B disease.

Adjuvant (postoperative) radiotherapy is well studied (Fletcher and Evers 1970; Snow *et al*, 1982; Vikram *et al*, 1984; Kramer *et al*, 1987). It decreases local failure rates and although only retrospective evidence exists (Huang *et al*, 1992), there is broad consensus since the 1980s that it increases survival and a randomised prospective trial would be unrealistic at this point. Still even with adjuvant radiotherapy, in the presence of high-risk features, the risk of local recurrence (27–61%), distant metastases (18–21%), and death (5-year survival rate 27–34%) remain unsatisfactorily high (Cooper *et al*, 1998).

Postoperative (adjuvant) CRT offered an approach that could enhance local control with radiosensitising chemotherapeutic agents. Several studies have demonstrated that concurrent CRT is a highly effective therapy for locally advanced HNSCC including tumours that are not amenable to surgery (El-Sayed and Nelson 1996; Brizel *et al*, 1998; Vokes *et al*, 1999; Jeremic *et al*, 2000; Adelstein *et al*, 2003; Vokes *et al*, 2003), justifying trials of concurrent CRT as postoperative (adjuvant) treatment.

The first trial to suggest a marked benefit of postoperative CRT over radiation alone in patients with locally advanced disease with high-risk features was a smaller trial by Bachaud *et al* published in 1996 (Table 2a) (Bachaud *et al*, 1996). In order to confirm the Bachaud trial, two similarly designed, multicentre, randomised phase III trials of adjuvant radiation *vs* CRT were reported in 2004 (Bernier *et al*, 2004; Cooper *et al*, 2004) also in patients with stage III, IVA/B disease and high-risk features for recurrence. The RTOG 9501 and the EORTC 22931 (summarised in Table 2) were able to produce level 1 evidence of a clear benefit for adjuvant CRT, at the cost of a significant increase in acute toxicities. Inclusion criteria and the type of radiotherapy varied somewhat between the two trials (Table 2), but overall they were coordinated to use similar treatment protocols, making the results appear very robust. Both trials support the benefit of CRT over radiation alone in patients with high-risk features. Although only the EORTC trial showed a significant survival advantage for CRT, the RTOG trial trended in the same direction and showed a significant increase in progression-free survival.

Also consistent in both trials was an increase in acute grade III and IV toxicities in the combined therapy arm (Table 2) including toxic deaths. On the other hand, there was no difference in severe long-term treatment-related toxicities. Strong consideration should, therefore, be given to treatment of these patients in centers with expertise in combined modality treatment and a well-established supportive care system. A positive correlation of treating institution expertise and patient survival supports this belief (Benasso *et al*, 1997).

Although we can conclude that postoperative CRT in patients with locally advanced disease with high-risk features is now standard of care (Table 2a), for patients without high-risk features, the evidence of a benefit of CRT over radiation alone is less clear with no randomised trials addressing this question. The Intergroup trial 0034 (Laramore *et al*, 1992) (see Table 2b) may give some insight, but clearly was not intended to address this particular question as it used sequential chemotherapy and radiotherapy in comparison to radiation alone. This trial did enroll a significant portion of intermediate risk patients (stage III disease) without high-risk features and did not find a difference in local control, disease-free, and overall survival. Interestingly, distal failures were decreased presumably due to the higher doses of systemic chemotherapy compared with concomitant CRT. Despite the enrollment of lower risk patients, overall local control rates were lower than in the more recent EORTC and RTOG trials. These trials were reported 12 years apart and make comparisons difficult due to stage migration and general improvements in supportive care and therapeutic modalities.

Even though adjuvant concomitant CRT as reported in these two landmark trials is a major step, it needs to be noted that the locoregional failure rate remains unsatisfactorily high at 30%. Attempts have been made to further improve the radiosensitising properties of chemotherapy using doublet and triplet combinations (see below) with the hope of further improving survival. Many centres have adopted this approach, based on phase II evidence indicating safety, feasibility, and potentially improved efficacy, but this remains an unanswered question until phase III data become available.

The optimal time frame to start adjuvant treatment postsurgery has not been studied sufficiently. Limited evidence and clinical experience with the time needed for patients to recover suggest that it should be within 4–6 weeks postsurgery.

### Definitive/concomitant CRT and organ preservation

During the past decade, an attractive alternative to initial surgery has evolved. Originally pioneered for inoperable patients, upfront concomitant CRT has emerged as a definitive treatment option comparable to upfront surgical management in resectable patients. Given its advantage with regards to organ preservation and excellent reported local control and survival rates, CRT is increasingly used and has become the dominant treatment modality in many centres (Hoffman *et al*, 2004). The decision between upfront surgery followed by chemoradiation *vs* upfront chemoradiation with the option of salvage surgery remains controversial and depends on many factors, including local expertise, goals for organ preservation, operability, resectability, and patient preference. No adequate randomised trial has examined this question and, given inherent biases in patient selection and ability to stage patients in a comparable fashion, it is unlikely that we will have a definitive answer in the near future. Both approaches work well, can coexist, and allow matching of treatments to a patient's disease and preferences.

#### Concomitant CRT

Concomitant CRT attempts to capitalise on radiosensitising properties while delivering systemically active agents. Sensitising effects though are not selective for tumour cells, and adjacent normal tissue within the field is also subject to more effective and more toxic radiation. Consistently, CRT trials report an increased incidence of grade 3 and 4 acute toxicities with mucositis and dermatitis being the most prominent. On the other hand, severe long-term side effects are not increased in comparison to radiation alone, and virtually all patients recover from the intense treatment. As mentioned, treatment should preferentially be done at experienced centres that have an appropriate support infrastructure (Benasso *et al*, 1997).

Multiple phase II trials using intensive CRT regimens have shown long-term survival rates of 60–70%, without surgery, for locally advanced HNSCC (Kies *et al*, 2001; Adelstein *et al*, 2002; Machtay *et al*, 2002; Vokes *et al*, 2003). Phase II trials need to be interpreted with caution due to their inherent biases, but the consistency of the results and the large number of patients treated with this approach is reassuring. Meta-analysis of early randomised, but mostly underpowered, trials suggested an absolute survival benefit of approximately 8% at 5 years for CRT over radiation also (NNT=13; 13 patients need to be treated to save one life) (El-Sayed and Nelson, 1996; Pignon *et al*, 2000). More recent trials furthermore suggest even more robust survival benefits with an absolute risk reduction of death at 3 years of 14–25% (NNT=4–7; between 4 and 7 patients need to be treated to save one life) (Brizel *et al*, 1998; Adelstein *et al*, 2003).

Based on suggestive phase II evidence, recent trials frequently now investigate combination chemotherapy. Commonly used agents include cisplatin, 5-FU (Table 2), taxanes, hydroxyurea, and gemcitabine (Vokes *et al*, 2003; Milano *et al*, 2004). Concomitant CRT is among the most efficacious locoregional control measures, but at the same time, these recent trials reveal a shift in the pattern of failure towards distant disease especially in patients with advanced nodal stage. Induction chemotherapy attempts to decrease distal failures has been advocated now that local control is achieved in most patients (see below).

Even though some controversy remains, there is an increasingly better defined role for surgical management of certain patients after CRT (Argiris *et al*, 2004). Cervical lymph node dissection (ND) even after a complete response (CR) to CRT is appropriate in patients with N2-N3 disease to optimise locoregional disease control (Lavertu *et al*, 1997; Argiris *et al*, 2004). Also, all patients with residual lymph node enlargement on imaging should undergo ND, even though many specimens will only show necrosis. In patient with higher LN status (N2 and higher), up to 35% of specimen will harbour residual microscopic tumour (Stenson *et al*, 2000). In a recent trial, this approach was able to improve progression-free survival (Argiris *et al*, 2004). In contrast, patients with N0-1 disease and a CR to treatment did not benefit. A selective lymph node dissection is feasible 4–12 weeks after CRT and is associated with an excellent safety profile (Stenson *et al*, 2000).

#### Organ preservation

Given that concomitant CRT increases locoregional control, and thereby avoids surgical resection of important anatomical structures, it was postulated that CRT may offer superior organ preservation in comparison to surgery, radiation, or sequential chemotherapy and radiation. Initially, two trials using sequential chemotherapy and radiation in comparison to surgery with adjuvant radiation reported improved organ preservation with no difference in survival (Table 3) (VA Laryngeal cancer study group, 1991; Lefebvre *et al*, 1996, 2004), establishing sequential treatment as a standard approach. At around the same time, CRT emerged as a highly efficacious treatment and a large Intergroup trial was therefore initiated. Intergroup trial 91–11 had as a primary end point larynx preservation in resectable patients with stage III or IV (but excluding T_4_) disease (Table 3) (Forastiere *et al*, 2003). The three treatment arms compared sequential chemotherapy and radiation, concurrent CRT, and radiotherapy alone. Laryngectomy was reserved for patients who did not respond to induction chemotherapy or with residual or recurrent disease following completion of radiotherapy on all three arms. Laryngeal preservation, the primary end point of this trial, was superior in the concomitant CRT arm compared to both sequential CRT (88 *vs* 75% 2-year laryngeal preservation rate, *P*=0.005) and radiotherapy alone (88 *vs* 70% 2-year laryngeal preservation, *P*<0.001). On the other hand, acute high-grade toxicities were more common in both arms involving chemotherapy. Similar to other CRT trials, late toxicities and swallowing function at 2 years were equivalent between the three arms. As a secondary outcome, CRT achieved the highest locoregional control rate. Survival was not significantly different between all three treatment arms, while both chemotherapy arms showed lower distant failure rates compared to radiotherapy alone. In conclusion concurrent CRT should be considered standard of care in this patient population. Sequential CRT is not more efficacious, but more toxic than radiotherapy, and should therefore not be routinely used.

In other anatomical locations, no comparable level 1 evidence for CRT exists. The earlier EORTC 24891 trial (Lefebvre *et al*, 1996, 2004) included hypopharynx tumours but only used sequential CRT. Still, based on the superior locoregional control rates in comparison to radiotherapy alone in locally advanced unresectable patients, CRT is likely to be at the very least a reasonable choice for organ preservation in general.

### An evolving role for induction chemotherapy

The administration of induction chemotherapy prior to definitive local therapy remains controversial. The interest in systemically active chemotherapy arose from the observation that, with highly effective local control measures, the majority of patients, who failed therapy, would recur at a distant site. This was presumably from micrometastatic disease that local therapy or lower dose chemotherapy as part of chemoradiation would not adequately treat. This theoretical argument has been tested in several trials that have been unable to show a consistent survival benefit (1987; Schuller *et al*, 1988; 1991; Paccagnella *et al*, 1994; El-Sayed and Nelson 1996; Domenge *et al*, 2000; Pignon *et al*, 2000). (Table 4a). Design issues as well as concerns that less effective local therapy in the past may have decreased the power to show a survival benefit, remain. Nevertheless, induction chemotherapy continues to be an ongoing area of research with several centres around the world continuing to investigate this approach, which can produce CR rates in 30–65% of patients and overall response rates of 70–85% (Hitt *et al*, 2003; Knecht *et al*, 2003; Knecht *et al*, 2003; Gustin *et al*, 2004; Nadeem *et al*, 2004; Vermorken *et al*, 2004).

Two European studies have shown evidence of a survival benefit with induction chemotherapy: the Italian GSTTC study (Gruppo di Studio sui Tumori della Testa) (Domenge *et al*, 2000) demonstrated increased cure rates in a subset of nonoperable patients and the French GETTEC trial (Groupe d'Etude des Tumeurs de la Tete et du Cou), which was closed early (Paccagnella *et al*, 1994). A large meta-analysis by Pignon *et al* (2000) furthermore showed a 5% increase in survival only for trials using a cisplatin/fluorouracil (5-FU) combination, reaching statistical significance (*P*=0.05).

More recently, two trials compared a triplet combination of a taxane (docetaxel or paclitaxel), cisplatin, and 5-FU (TPF) with doublet cisplatin/5-FU (PF) (Hitt *et al*, 2003; Vermorken *et al*, 2004). Consistent in both randomised trials, TPF was well tolerated and significantly more effective in particular in the recently reported EORTC 24971 trial by Vermorken *et al* (2004), where a significant difference in overall survival was seen. The trials are not fully published, but the data from both trials are consistent suggesting that a triplet combination (TPF) including a taxane has potential to emerge as a standard choice for induction chemotherapy in the future.

Still induction chemotherapy cannot be considered standard of care due to the lack of convincing phase III evidence, but lower level evidence suggests that it is reasonably safe and may benefit patients at high risk for distal failure as indicated by advanced nodal involvement. The triplet combination of a taxane, cisplatin, and 5-FU seems to have a high degree of activity and acceptable toxicity (Table 4b) (Hitt *et al*, 2003; Knecht *et al*, 2003; Vermorken *et al*, 2004).

#### Induction chemotherapy and concomitant CRT

The combination of induction chemotherapy and concomitant CRT appears to be of particular interest due to their complementary effects with the former leading to a reduction of distant disease and the latter achieving locoregional control. Results of induction chemotherapy followed by CRT are encouraging (Hainsworth *et al*, 2002; Machtay *et al*, 2002; Haraf *et al*, 2003; Vokes *et al*, 2003) (Table 4b) and suggest a reasonable toxicity profile, lower distal failure rates, and improved survival in comparison with historical controls using the same CRT regimen (Vokes *et al*, 2003).

In summary, there is a possibility that induction chemotherapy may improve survival in locally advanced HNSCC in combination with CRT as suggested by lower level evidence. Adequate phase III evidence is not available currently, and until further data become available, induction chemotherapy should not be used routinely in HNSCC outside a clinical trial, but may be appropriate given its well-established safety profile in high-risk situations.

### Epidermal growth factor receptor inhibition in combination with radiotherapy or CRT

The EGF receptor as well as its principal ligand TGF-*α* are expressed in approximately 80% of HNSCC (Grandis *et al*, 1996; Grandis *et al*, 1998) and play an important role in the biology of this disease (Barrandon and Green 1987; Grandis *et al*, 1998; O-charoenrat *et al*, 2000; Veikkola *et al*, 2000; Ang *et al*, 2002). A multitude of agents inhibiting EGFR are in various stages of development and encouraging single agent activity has been reported in recurrent or metastatic HNSCC (Cohen EE *et al*, 2003; Soulieres *et al*, 2004). Despite the high efficacy of current treatments, EGFR inhibitors hold promise in two important ways: (1) to further improve efficacy for patients at risk for recurrence and (2) to decrease treatment-related toxicities by replacing more toxic cytotoxic drugs without jeopardising survival.

A phase III multicentre trial (Bonner *et al*, 2004) enrolled 424 patients with stage III/IV HNSCC randomising them to receive radiotherapy with or without concomitant cetuximab, a humanised monoclonal antibody directed against the EGFR, as a primary treatment. Overall, the regimen was well tolerated, with an increase in grade 3 cutaneous toxicity from 18 to 34% and some mild increases in allergic reactions. Even though only preliminary data are available, a marked increase in median overall survival from 28 months with radiation alone to 54 months with the addition of cetuximab was reported. Locoregional control was significantly improved in the cetuximab arm as well (48 *vs* 56% at 2 years, *P*=0.02).

These reports are very encouraging; however, the major difficulty in interpreting and using these data is that it remains unclear how radiotherapy plus cetuximab compares to CRT as the reported trial used a control arm that would be considered inferior in light of recent evidence discussed above. Given the immature nature of the data and in particular the inability to compare to current standards of care, it is prudent to wait until further information becomes available. Still it is reasonable to consider this regimen in patients with poor performance status, who are not good candidates for CRT or surgery.

Another report of the combination of gefitinib with CRT (5-FU, hydroxyurea, and twice daily radiotherapy), following induction chemotherapy and followed by gefitinib maintenance, provided insights into a potential role for EGFR inhibitors (Cohen EW *et al*, 2004). In comparison to previous similarly designed trials, this treatment was at least as efficacious as a comparable taxane-containing regimen (paclitaxel, 5-FU, hydroxyurea, and radiotherapy) (Vokes *et al*, 1999) and better tolerated. Another trial exploring the combination of gefitinib and radiation with or without cisplatin is currently ongoing and demonstrates that the combination of gefitinib with cisplatin and radiotherapy appears feasible.

## CONCLUSION

**Figure 1:**
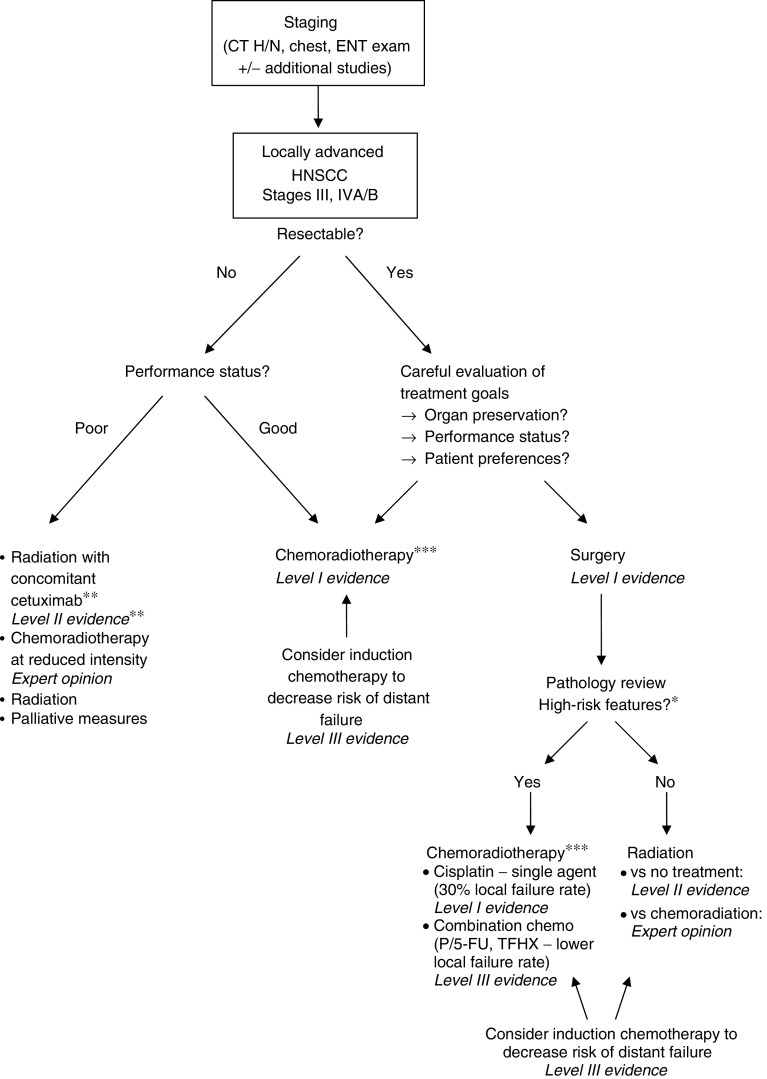
Evidence-based treatment algorithm for management of locally advanced HNSCC.

Treatment for locally advanced HNSCC has improved dramatically during the past decade, allowing a discussion of cure in many patients, and an evidence-based algorithm to guide treatment can be created (Figure 1). In particular, CRT has established itself as a central treatment modality either upfront as definitive therapy or as an adjuvant to surgery, due to its excellent local control rates, increased survival, and higher rates of organ preservation. For the future, the integration of EGFR inhibitors is poised to play an increasingly important curative role while potentially decreasing toxicity. Still it may be even more important to consolidate our current knowledge and abilities and enforce quality standards to allow as many patients as possible to benefit from already excellent treatment approaches.

## Figures and Tables

**Table 1:**
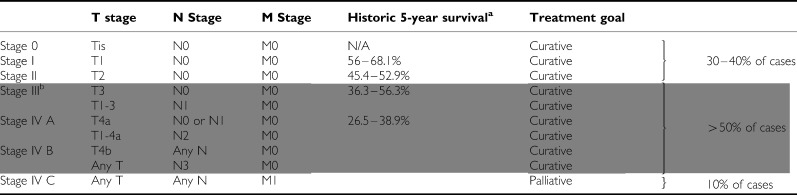
Staging overview, for details of T and N staging, please refer the AJCC staging manual

**Table 2a:** Postoperative therapy: randomised trials of adjuvant chemoradiotherapy *vs* radiotherapy alone in locally advanced HNSCC

**Trial name Year** **reported**	**No of patients**	**Primary** **treatment**	**Adjuvant therapy**	**Toxicities – grades** **3 and 4**	**Higher local** **control rate**	**Survival difference** **in favour of** **ChemoRT**
EORTC^a^ 22931 (Bernier *et al*, 2004)	167 with high-risk features	Surgery	*ChemoRT (P)* RT^b^	Acute: *41* *vs* 21% Chronic: no difference	Yes *82* *vs* 69% (at 5 years)	OS: Yes, HR=0.7, *P*=0.02 DFS: Yes, HR=0.75, *P*=0.04
RTOG^a^ 9501 (Cooper *et al*, 2004)	459 with high-risk features	Surgery	*ChemoRT (P)* RT^b^	Acute: *77 vs* 34% Chronic: no difference	Yes *82* *vs* 72% (at 2 years)	OS: No, HR=0.84, *P*=0.19 DFS: Yes, HR=0.78, *P*=0.04
Bachaud *et al* (1996)	83 with high-risk features	Surgery	*ChemoRT (P)* RT	Acute: *41 vs* 18% Chronic: no difference	Yes *77* *vs* 59% (at 4 years)	OS: Yes DFS: Yes

EORTC=European Organization for Research and Treatment of Cancer; RT=radiotherapy; ChemoRT=concurrent chemoradiotherapy; OS=overall survival; DFS=disease-free survival; *vs*=versus; HR=hazard ratio; high-risk features=please refer to paragraph on adjuvant, postoperative therapy.

a Inclusion criteria at 4 years, not significant; *P*=Cisplatin. *Inclusion criteria* *EORTC*: The EORTC study defined four eligible groups of patients: (1) pathologically proven T3 or T4 primary tumours with any nodal stage (N), except T3N0 completely resected laryngeal cancers (negative resection margins); (2) pT1 or pT2 tumours with an N2 or N3 nodal stage (M0); (3) patients with T1/T2 primary tumours, N0/N1 nodal status but unfavourable pathological findings such as extranodal spread, positive resection margins, perineural involvement, or vascular tumor embolism; (4) oral cavity or oropharyngeal tumours with involved lymph nodes at levels IV or V (lower jugular area and posterior neck triangle (Robbins *et al*, 1991). *RTOG*: any or all of the following features needed to be present: (1) histologic evidence of invasion of two or more regional lymph nodes; (2) extracapsular spread of nodal disease; (3) microscopically involved mucosal resection margins.

b *Radiotherapy regimens* *EORTC*: up to 54 Gy in 27 fractions to a large volume including all tumour sites over a period of 5 1/2 weeks. High-risk areas (risk for dissemination, inadequate resection margins) received a ^[6]^12-Gy boost (→ total 66 Gy; 33 fractions over a period of 6 1/2 weeks). *RTOG*: 60 Gy in 30 fractions over a 6-week period, with or without a boost of 6 Gy in three fractions over a period of 3 days to high-risk sites.

**Table 2b:** Postoperative therapy: randomised trial of adjuvant sequential chemotherapy+radiotherapy *vs* radiotherapy alone in locally advanced HNSCC

**Trial name Year** **reported**	**No of patients**	**Primary** **treatment**	**Adjuvant therapy**	**Toxicities – grades** **3 and 4**	**Higher Local** **Control rate**	**Survival difference** **in favour of** **Chemo+RT**
Intergroup 0034 (Laramore *et al*, 1992)	442 mix of intermediate and high- risk patients	Surgery	*Sequential Chemo* *(PF)*+*RT* RT	Acute: no difference Chronic: no difference	No *74* *vs* 71% (at 4 years)	OS: No *48* *vs* 44%^a^; DFS: No *46* *vs* 38%^a^ → Lower distal failure rate; 15 *vs* 23% (at 4 years)

RT=radiotherapy; Chemo=chemotherapy (cisplatin based); OS=overall survival; DFS=disease-free survival; *vs*=versus; high-risk features=please refer to paragraph on adjuvant, postoperative therapy.

a At 4 years, not significant; PF=cisplatin + 5-FU.

**Table 3:**
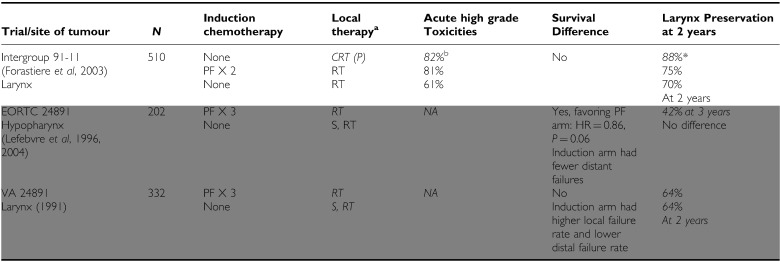
Chemoradiotherapy *vs* other modalities for organ preservation in locally advanced HNSCC

**Table 4a:** Randomized trials of induction (Neoadjuvant) chemotherapy *vs* no induction chemotherapy in locally advanced HNSCC

**Trial**	***N***	**Induction chemotherapy**	**Local therapy**	**Survival difference**	**Lower distant failure**
HNCP (Head & Neck Contracts Program, 1987)	462	PB X 1 PB X 1, P X 6^a^ None	S, RT S, RT S, RT	No	Yes, arm B
SWOG (Schuller *et al*, 1988)	158	PMBV X 3 None	S, RT S, RT	No	No, *P*=0.07
GSTTC (Paccagnella *et al*, 1994)	237	PF X 4 None	S, RT^b^ S, RT	Yes, for inoperable patients only	Yes
GETTEC (Domenge *et al*, 2000)	318	PF X 3 None	S, RT^b^ S, RT	Yes	No

HNCP=head and neck contracts program; SWOG=southwest oncology group; GSTTC=Gruppo di Studio sui Tumori della Testa e del Collo; GETTEC=Groupe d'Etude des Tumeurs do la Tete et du Cou; RT=radiotherapy; S=surgery; P=cisplatin; F=5-fluorouracil; B=bleomycin; M=methotrexate; V=vincristine; N/A=not applicable.

a Arm B of the HNCP trial administered one cycle of induction and six cycles of adjuvant chemotherapy.

b Patients on the GSTTC and GETTEC studies received local therapy based on operability: operable patients received surgery and adjuvant RT; inoperable patients received 65–70 Gy RT.

**Table 4b:** Randomized trials of comparing PF *vs* TPF induction (neoadjuvant) chemotherapies

**Trial**	***N***	**Chemotherapy**	**Local Therapy**	**Survival Difference**	**Toxic deaths**
Hitt *et al* (2003)	383	PF X 3 TPF X 3 (T=paclitaxel)	CRT (P) CRT (P)	Yes, HR=0.79 in favour of TPF; *P*=0.038	PF: 4.1% TPF: 2.1% NS
EORTC 24971 (Vermorken *et al*, 2004)	358	PF X 4 TPF X 4 (T=docetaxel)	RT RT	Yes, HR=0.73 in favor of TPF; *P*=0.016	PF: 5.5% TPF: 2.3% Significant

EORTC=European Organization for Research and Treatment of Cancer; CRT=chemoradiotherapy; RT=surgery; P=cisplatin; F=5-fluorouracil; T=taxane; N/A=not applicable; NS=not significant; HR=hazard ratio.
